# Electrophilically Activated Nitroalkanes in Reactions With Carbon Based Nucleophiles

**DOI:** 10.3389/fchem.2020.00077

**Published:** 2020-02-11

**Authors:** Nicolai A. Aksenov, Alexander V. Aksenov, Sergei N. Ovcharov, Dmitrii A. Aksenov, Michael Rubin

**Affiliations:** ^1^Department of Chemistry, North Caucasus Federal University, Stavropol, Russia; ^2^Department of Chemistry, University of Kansas, Lawrence, KS, United States

**Keywords:** nitroalkanes, electrophilic attack, synthetic methodology, C-H functionalization, oximes

## Abstract

Unusual reactivity of nitroalkanes, involving formation of aci-forms (nitronic acids or nitronates) and their subsequent interaction with carbon-based nucleophiles, is surveyed in this review.

## Introduction

Aliphatic nitro compounds can be easily deprotonated at α-position in the presence of bases, and the formed anionic intermediates are normally employed in a variety of nucleophilic addition or substitution reactions. However, the same compounds can also serve as electrophilic synthons after Bronsted acid-assisted tautomerization into aci-forms. In these form nitro-compounds can be attacked, for example by electron rich arenes to form aryloximes, which can be further transformed into anilides (Yadav et al., 2010; Augustine et al., 2011), anilines (John and Bergens, 2011), ketones (Joseph et al., 1994), aldehydes (Ranu and Sarkar, 1988), or various products of their subsequent rearrangements (Sardarian and Shahsavari-Fard, 2008). It should be pointed out, that *in-situ* generated aci-form is only weakly nucleophilic and can efficiently interact with a limited number of relatively strong nucleophiles. This process could be severely impeded by unfavorable position of the tautomeric equilibrium, which is normally shifted toward nitro-form at low pH. This situation can be addressed by carrying out the reaction in the presence of excess acid at low temperature as in classical Nef reaction (Nef, 1894). To this end, strong Bronsted (Nakamura et al., 2007) or Lewis (Marce et al., 2016) acids could be employed. An alternative approach involves a tying the reactive aci-tautomer in acylated (Lehr et al., 1979), alkylated (Falck and Yu, 1992), or silated (Colvin et al., 1980) forms. Analysis of synthetic methods employing these unusual electrophilic synthons and their application in preparation of medicinally relevant scaffolds are analyzed in this review.

**Scheme 1:**
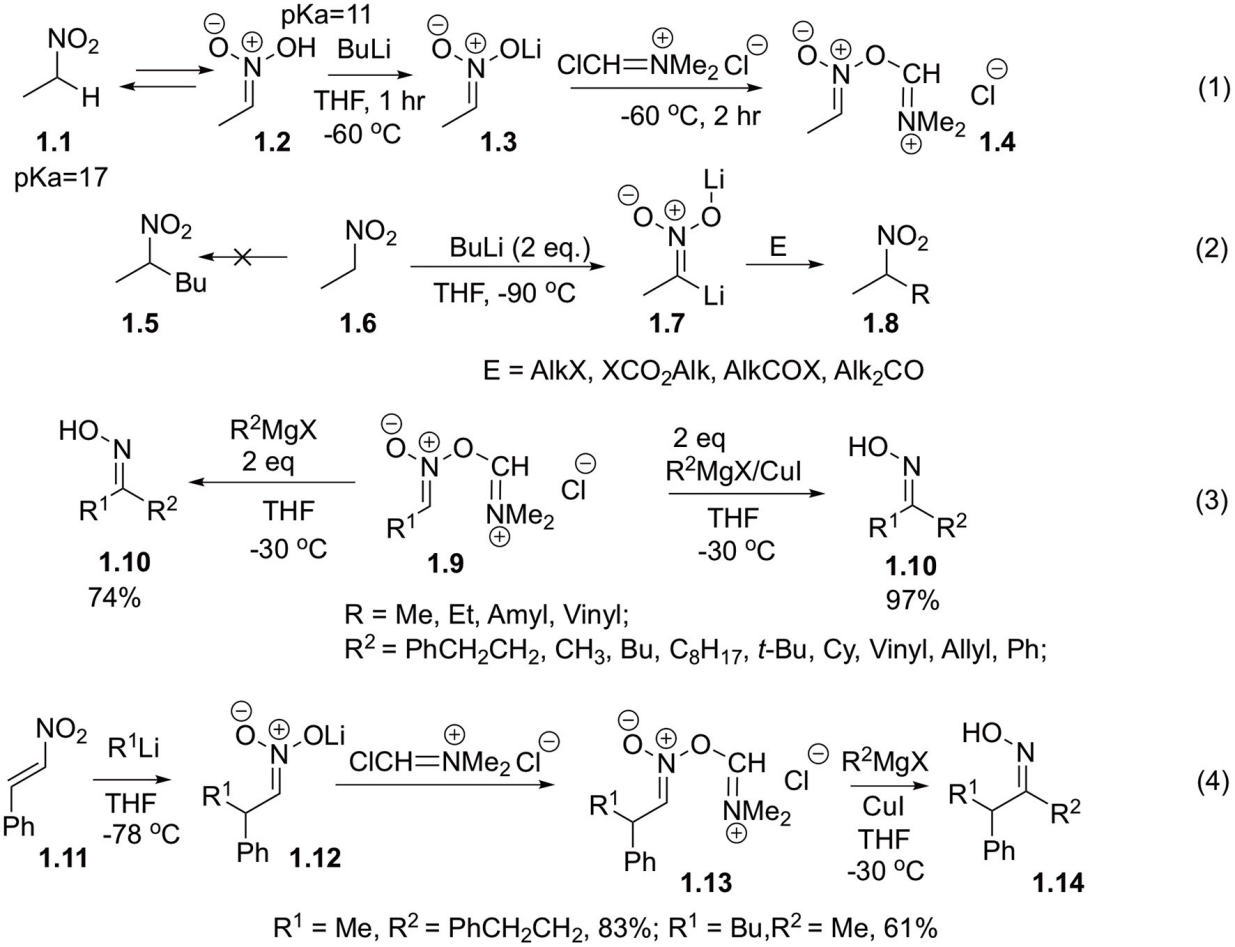
Reactions of electrophilically activated nitroalkanes with Vilsmeier reagent.

Processes of C-Aryl bonds formation with participation of nitroalkanes in a role of electrophilic synthon are pretty exotic. The reasons for this are summarized in our introduction and were very well-detailed in several reviews (Lalli, 2000; Smirnov et al., 2012; Tabolin et al., 2017, 2018). Arguably, one of the earliest contributions to this area was made by Fujisawa, who demonstrated interaction of nitroalkanes with organometallic reagents (Fujisawa et al., 1983). To this end, nitroethane **1.1** was treated with *n*-BuLi and then with Vilsmeier reagent (Scheme 1, Equation 1). It is known that lithium nitronate derivatives are not electrophilic, so even in the presence of excess *n*-BuLi they only afford products of 2-fold deprotonation. These dianions (**1.7**) can be employed as nucleophilic synthons in reactions with ketones (Seebach et al., 1982), as well as alkylating (Seebach et al., 1977) or acylating (Ram and Ehrenkaufer, 1986) reagents (Scheme 1, Equation 2). Interestingly, the generated aci-nitroiminium chloride species **1.9** could also be involved in the reaction with two equivalents of Grignard reagent to afford oximes **1.10** in good yields (Fujisawa et al., 1983). Catalytic version of this reaction was also investigated, to demonstrate that in the presence of copper(I) chloride the same oximes (**1.10**) were generated in nearly quantitative yields (Scheme 1, Equation 3) (Fujisawa et al., 1983). It should be pointed out that nitronates **1.12** could be generated *in situ* via addition of lithiumorganic compounds to *trans*-β-nitrostyrenes **1.11** and further reacted with Grignard reagents in the same manner (Scheme 1, Equation 4) (Fujisawa et al., 1983).

**Scheme 2:**
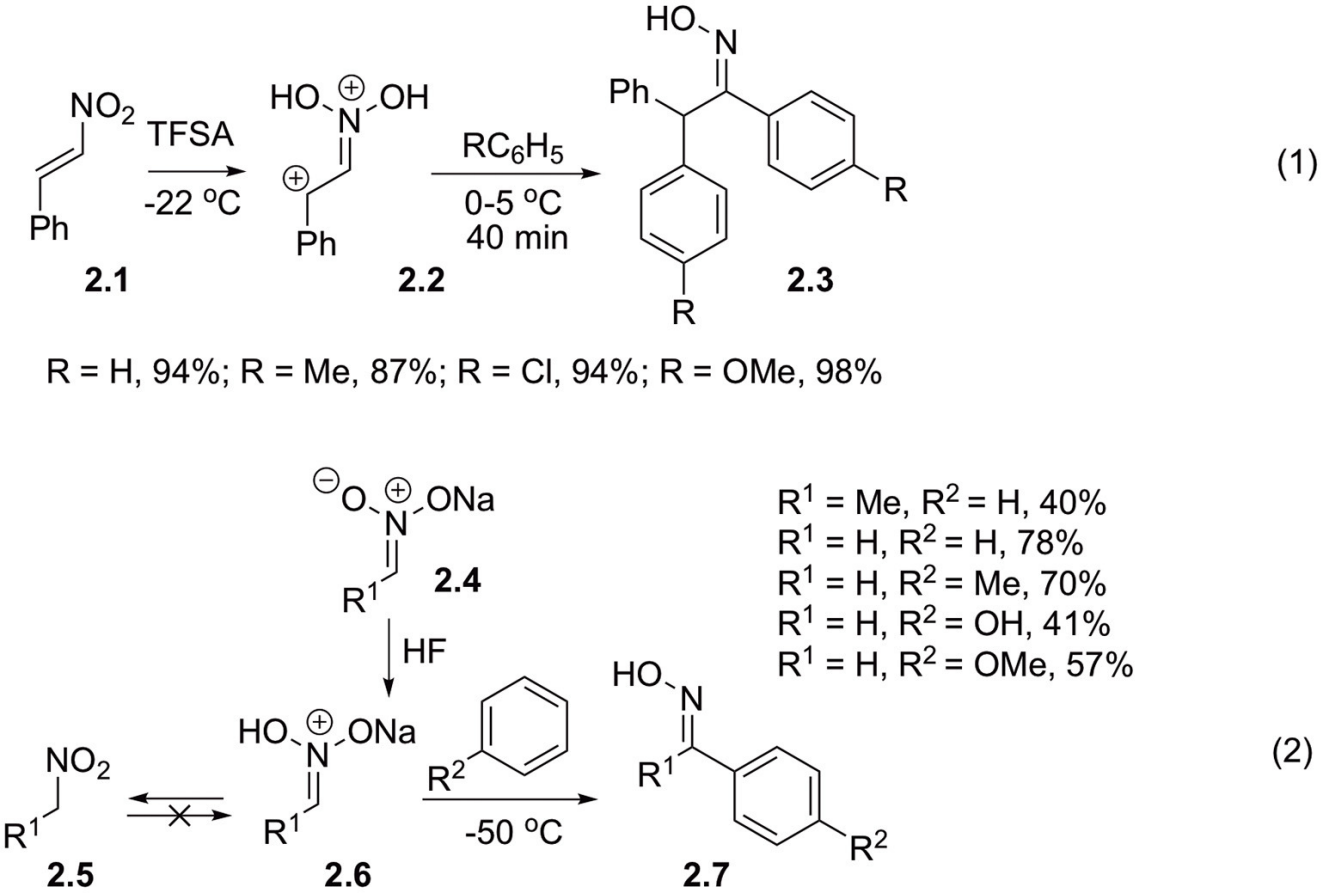
Alpha-activation of nitroalkanes toward reaction with electron-rich arenes.

Much more interesting are synthetic routes, involving direct interaction of nitroalkanes with nucleophilic arenes. Pioneering research in this area was performed by Ohwada et al. (1987a,b). He discovered that β-nitrostyrenes **2.1** in triflic acid form *N, N*-dihydroxyiminiumbenzyl dications **2.2**, which was confirmed by a series of cryoscopic spectral methods, including ^18^O-labeling studies (Ohwada et al., 1986). These electrophiles are sufficiently reactive to be intercepted even by chlorobenzene. Nitronic acid formed in this process subsequently reacts with a second equivalent of arene affording 1,2,2-triarylethan-1-one oxime **2.3** (Scheme 2, Equation 1). Beckmann rearrangement of this oxime did not occur, probably, under highly acidic conditions it exists in diprotonated form. Influence of diprotonation on reaction yields was investigated in details. Thus, it was shown that upon employment of trifluoroacetic and sulfuric acids as well as in the presence of substoichiometric amounts of trific acid, the reaction afforded diarylation products in marginal yields (c.a. 30%), which was explained by low concentration of the diprotonated form in these media. Later, it was discovered that protonated nitronic acids could also be involved in reactions with arenes. To this end sodium nitronate **2.4**, obtained from nitromethane or nitroethane was slowly added to a cold mixture of benzene and HF (Scheme 2, Equation 2). The authors pointed out that the reaction yields of oximes **2.7** were affected by a number of factors, and that the average reaction efficiency is related to competing non-productive C-protonation, which produce non-reactive nitroalkanes. This correlates well with trends observed in mechanistically related Nef reaction.

**Scheme 3:**
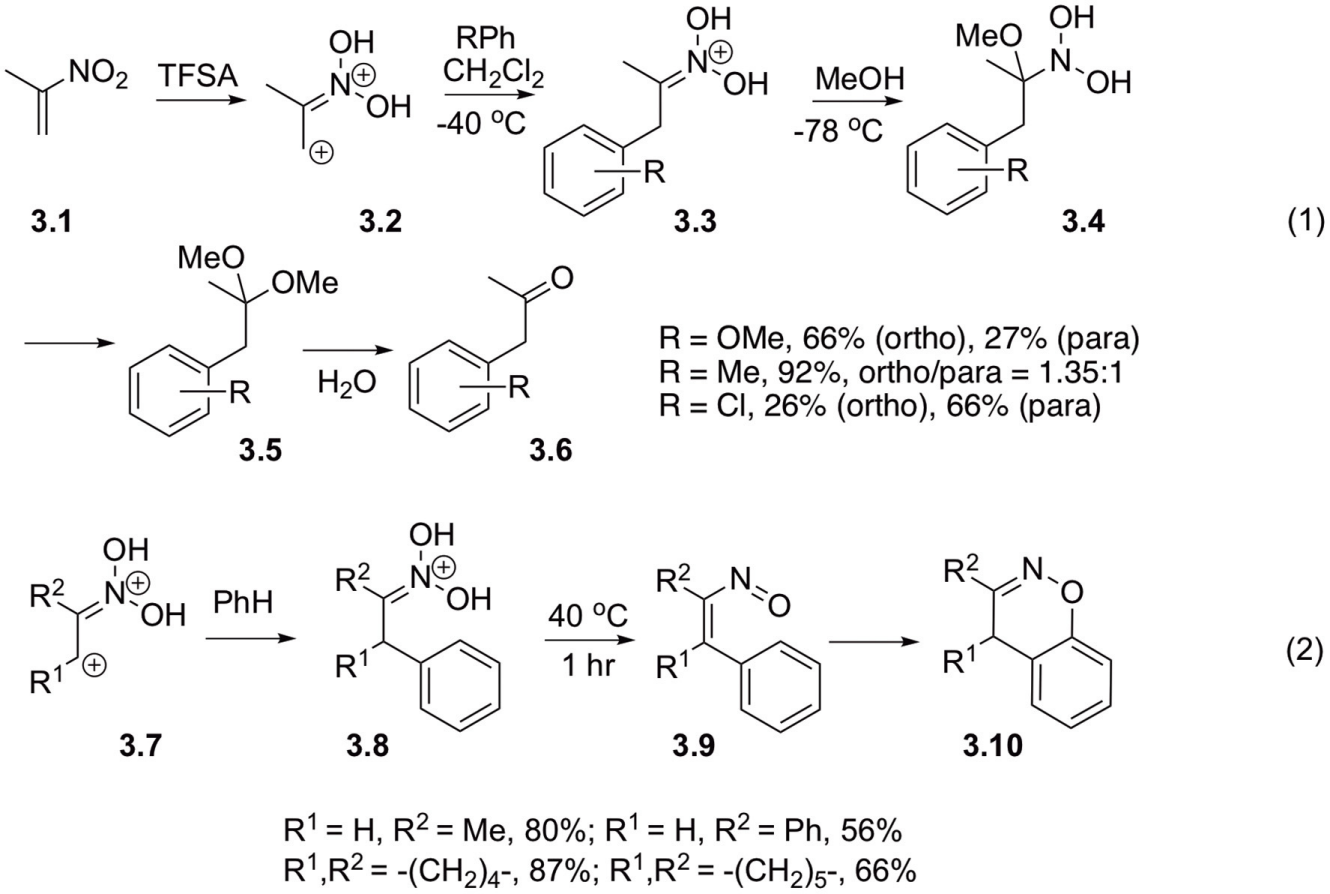
Beta-activation of nitroalkanes toward reaction with electron-rich arenes.

Ohwada with co-workers also investigated reactions of diprotonated 2-nitropropenes **3.1** with nucleophilic arenes. It was demonstrated, that in the presence of methanol aci-form **3.3** formed after Michael addition of arene to nitroalkene, could be efficiently involved in the Nef reaction, ultimately providing arylacetone **3.6** (Scheme 3, Equation 1) (Okabe et al., 1989; Ohwada et al., 1990). It should be pointed out, that this reaction might take a different route at elevated temperatures. Typically, in this case, nitronate **3.8** formed after addition of arene species to nitroalkene undergo rearrangement into unsaturated nitroso compound **3.9**, which subsequently cyclizes into 1,2-benzoxazine **3.10** (Scheme 3, Equation 2) (Ohwada et al., 1990).

**Scheme 4:**
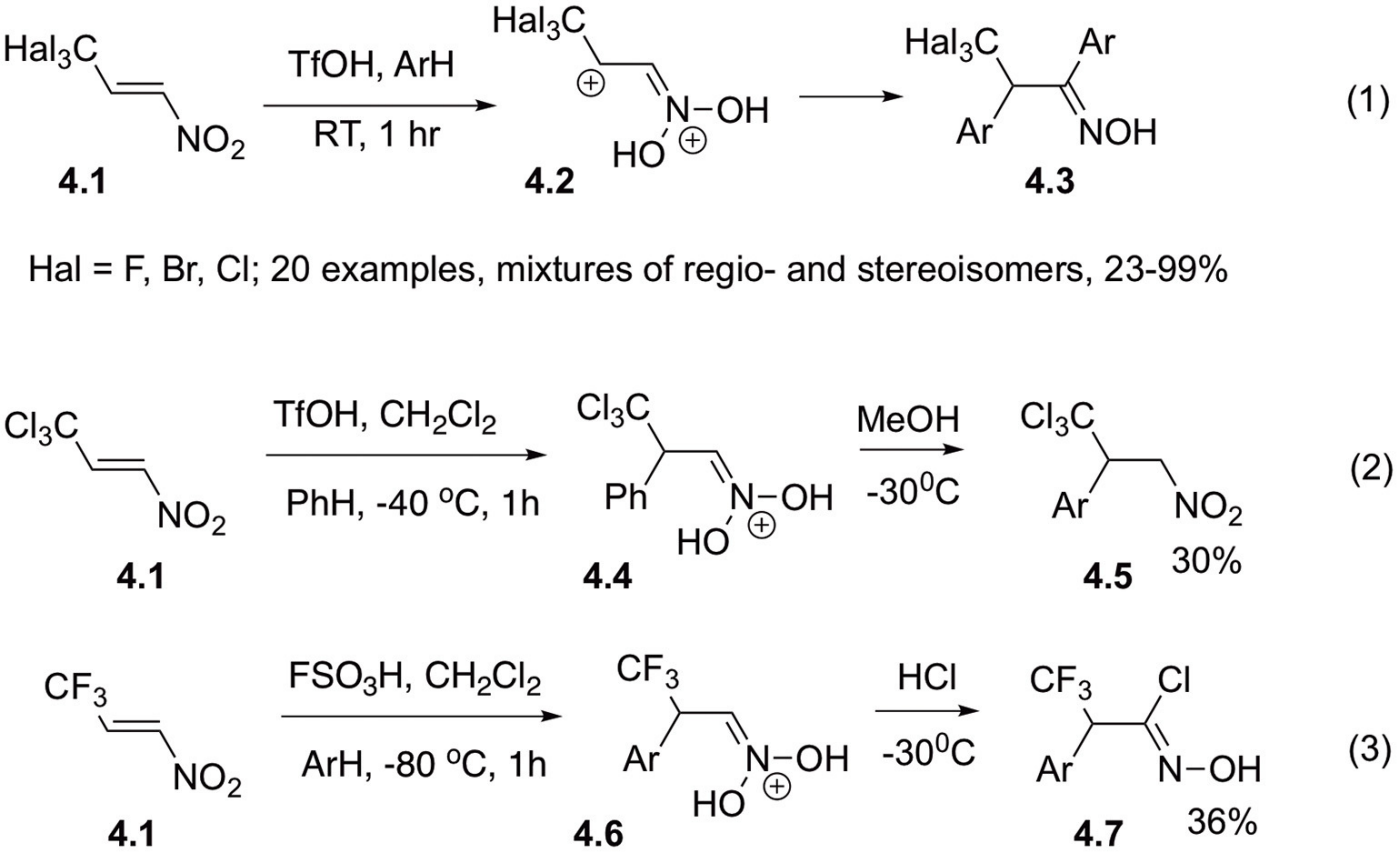
Reaction of trihalogenmethylated nitro-olefins.

Synthetic strategy capitalizing on double arylation of nitroalkenes was further developed in recent work by Golushko et al. (2018), describing reaction of 2,2,2-trihalogeno-1-nitroethylenes **4.1** with arenes in the presence of strong Brønsted and Lewis acids (Scheme 4, Equation 1). The best yields of bis-arylated products **4.3** were obtained in the presence of excess TfOH at room temperature. The reaction intermediates were studied by NMR spectroscopy and computer modeling. Regioselectivity of the reaction is not high and, typically, mixtures of regioisomers are formed. In reaction of sterically encumbered CBr_3_-derivatives high para-selectivity was achieved for the first (Michael reaction) step. Also, it should be pointed out that the products of *ortho*-substitution are forming mixtures of atropomers with axial chirality, which complicates analysis of the product mixture even further. An additional support for the reaction mechanism is obtained by intersection of the intermediate cations **4.6** with an appropriate nucleophile, for example chloride ion (Scheme 4, Equation 3). Under the same conditions, methanol only serves as a proton carrier and mediates isomerization of the aci- into nitro-form **4.5** (Scheme 4, Equation 2) (Golushko et al., 2018).

**Scheme 5:**
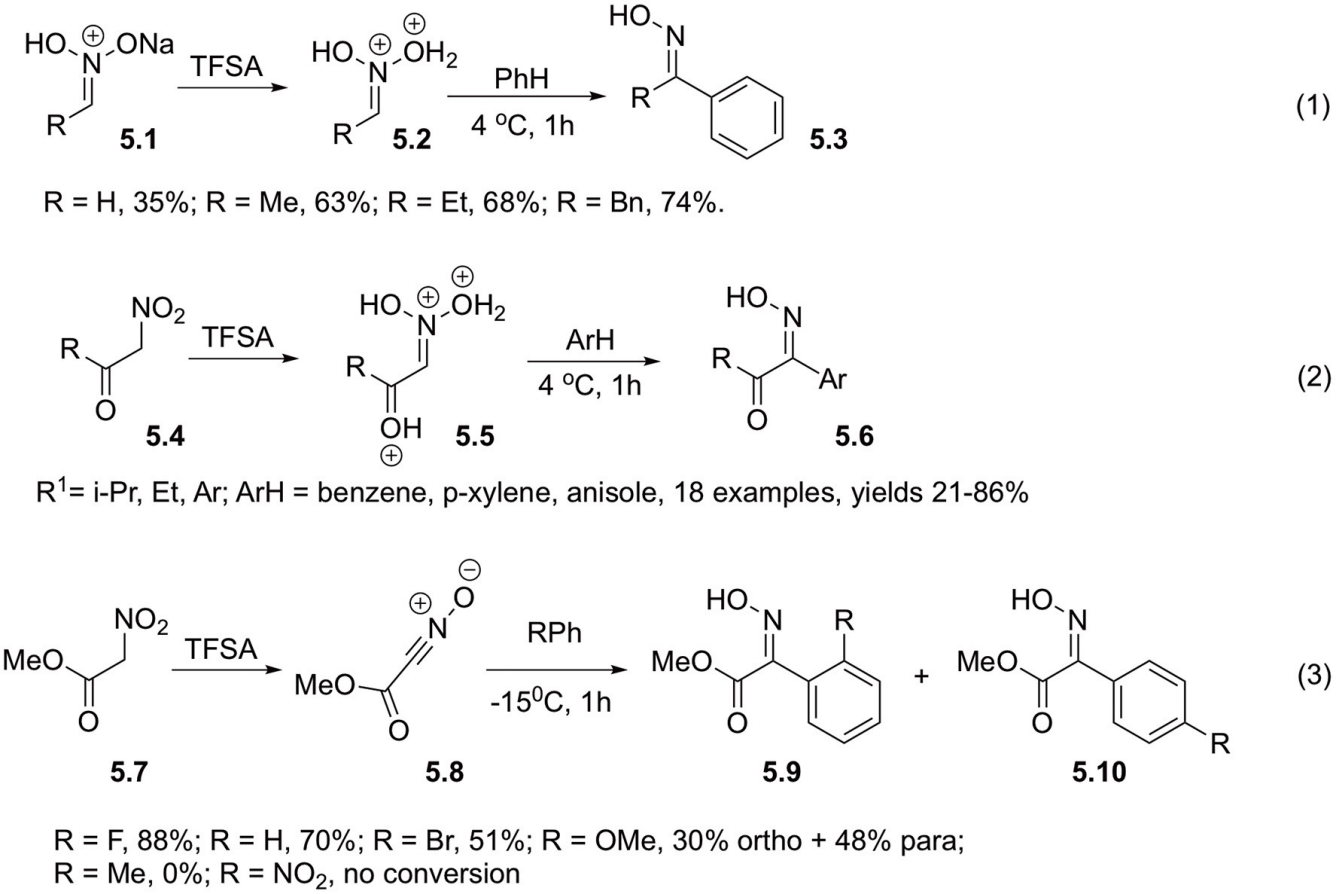
Employment of TFSA to produce aci-form of nitroalkanes.

Reaction of nitroalkanes in aci-form (**5.1**) with non-activated arenes also occur in the presence of triflic acid and provides the corresponding oximes **5.3**. In this case, participation of bis-protonated form of nitronic acid **5.2** was also postulated (Scheme 5, Equation 1). Utilization of 10% solution of triflic acid in TFA allows to improve the reaction efficiency (Ohwada et al., 1991). It should be pointed out, that benzoylnitromethanes **5.4** readily enolizable in the presence of strong Brønsted acid could participate in this type of transformations directly, without need for a separate conversion into nitronate form (Scheme 5, Equation 2). Computations suggest that reactive electrophilic species in this transformation is triple-protonated nitroketone species **5.5**. This reaction was studies for a wide range of substrates, and typically provides good yields of products **5.6** derived from benzene, xylenes and anisole. In the latter case, however, *para*/*ortho* selectivity is quite low, providing mixtures of products c.a 2:1 (Scheme 5, Equation 2) (Ohwada et al., 1991; Takamoto et al., 2009).

Nitroacetic esters **5.7** also react under the same conditions (Coustard, 1991; El Bahhaj et al., 2014). At 15°C they were shown to react with halogen- and alkoxy-substituted arenes affording oximes of α-ketoesters **5.9** and **5.10** in high yields (Scheme 5, Equation 3). These compounds were used as key intermediates in preparation of psammapline-like inhibitors of histone deacetylase. In contrast to Ohwada (Ohwada et al., 1991), advocating for formation of multi-protonated nitronates, Jacquesy in his work postulated formation of nitrile oxide **5.8** as a key electrophilic intermediate (Scheme 5, Equation 3) (Coustard et al., 1992); although this hypothesis was not experimentally verified.

**Scheme 6:**
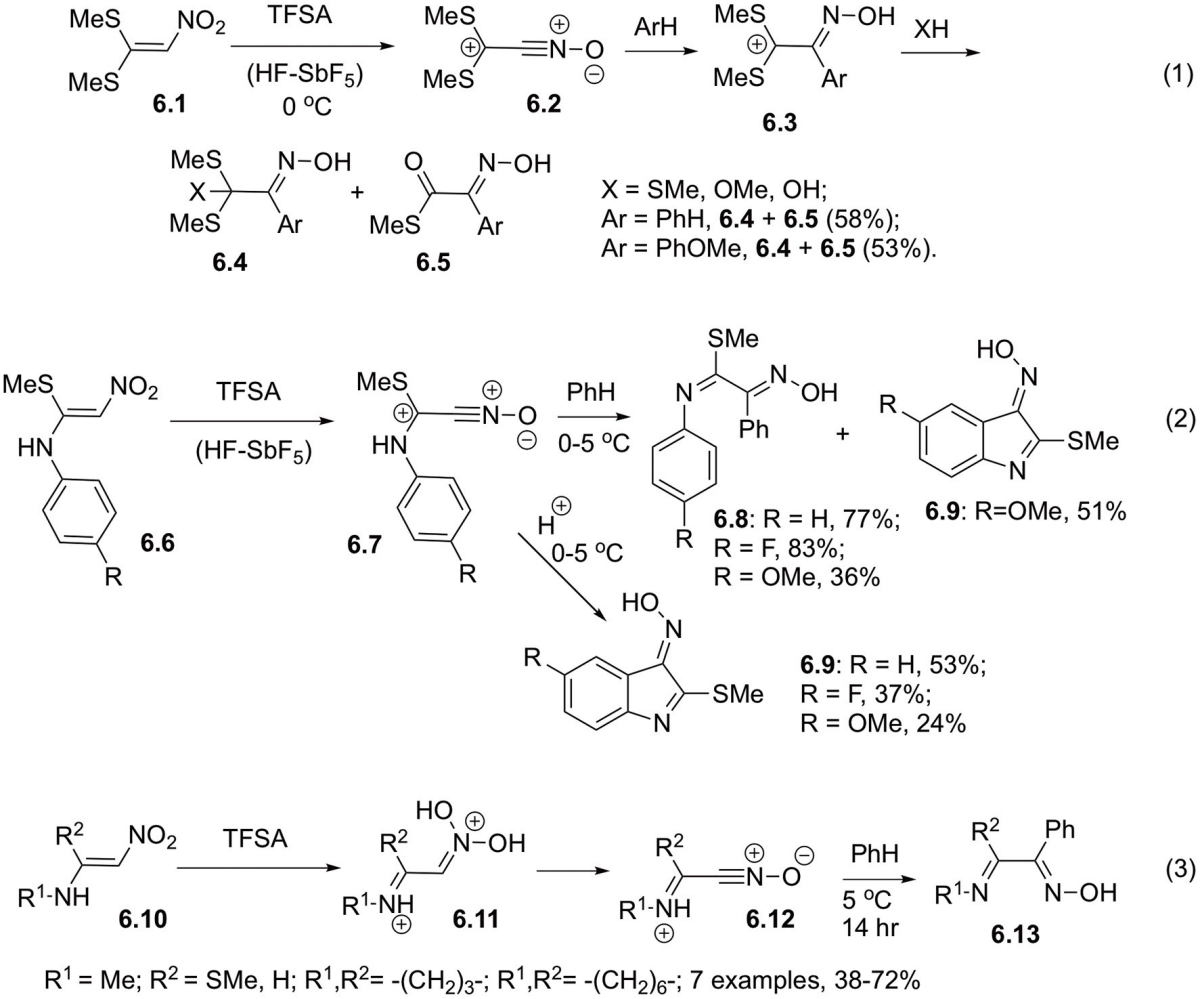
Reactivity of 1,1-bis(methylthio)-2-nitroethylene.

It was shown that 1,1-bis(methylthio)-2-nitroethylene (**6.1**) could be transformed into the corresponding nitrile oxide **6.2** in superacidic medium (triflic acid or HF-SbF_5_). This nitrile oxide can be intercepted with various nucleophilic reagents, including electron rich arenes. Structures of products **6.3** and intermediates were investigated and the proposed mechanism was supported with NMR-spectroscopy and mass-spectrometry (Scheme 6, Equation 1). It should be pointed out, that high *para*-selectivity was observed in reactions involving anisole (Coustard and Jacquesy, 1993; Coustard, 1995). Similarly, at low temperature in superacidic medium proceeds diprotonation of 1-arylamino-1-methylthio-2-nitroethylenes **6.6**. Subsequent elimination of water leads to the formation of nitriloxides **6.7**, which could be intercepted with benzene to afford arylimino derivatives **6.8** (Scheme 6, Equation 2). Since the starting material **6.6** employed in this reaction contained aniline moiety, at higher temperature intramolecular cyclization occur to produce indole core **6.9** (Scheme 6, Equation 2) (Kearney et al., 1992b; Coustard, 1996; Cousson and Coustard, 1998). For better understanding of this process, reactivity of *N*-alkyl derivatives of 1-amino-2-nitroethylene **6.10** was also studied. It was confirmed, that in solution of triflic acid these substrates experience C,O-diprotonation to produce hydroxynitrilium ions **6.11**, which could further react either with triflate anion or with benzene serving as a solvent. Reaction with triflate is a major detrimental factor affecting the efficiency of formation of the target oxime molecules **6.13** (Scheme 6, Equation 3) (Coustard, 1999).

**Scheme 7:**
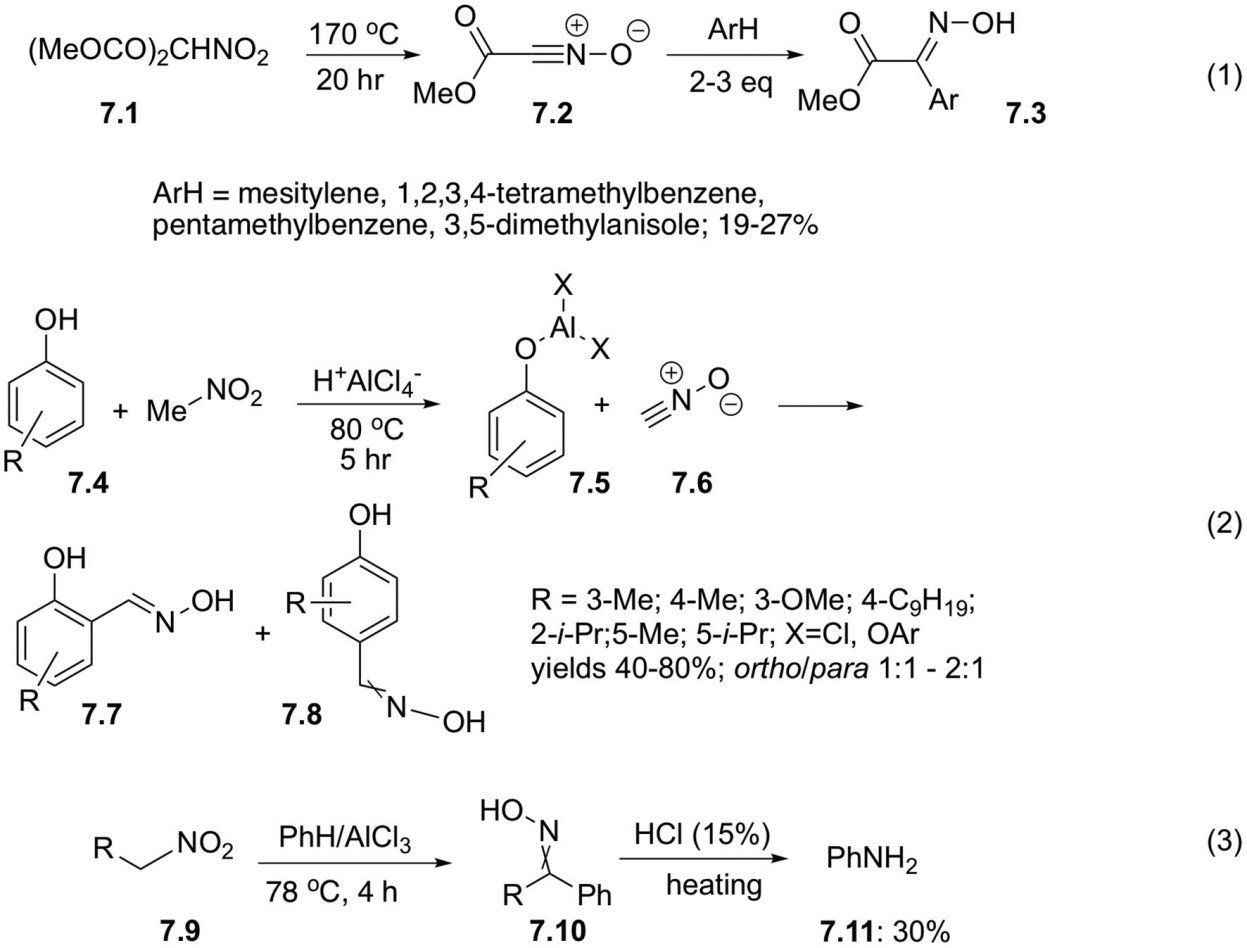
Reactions of nitroalkanes with arenes under conditions of Frieden-Crafts reaction.

Decarboxylation reaction could serve as a great alternative to α-deprotonation in the process of generation of aci-forms of nitroalkanes. Upon heating, dimethylnitromalonic ester **7.1** undergoes decarboxylation with subsequent elimination of water from nitro-functionality. The resulting methylcarbonocyanidate *N*-oxide **7.2** could undergo further dimerization into bis(carbomethoxy)furoxan or could be intercepted with dipolarophiles of electron rich aromatic compounds to afford oximes **7.3** in marginal yields. It should be mentioned that this reaction is limited to sterically hindered arene substrates, such as mesitylene, 1,2,3,4-tetramethylbenzene, pentamethylbenzene, or 3.3-dimethylanisole (Scheme 7, Equation 1) (Shimizu et al., 1985).

Satori and co-workers suggested that nitriloxides generated from nitroalkanes could react with phenoxides to afford salicylic oximes. Indeed, this reaction of phenols **7.4** involving nitromethane proceeds well in the presence of AlCl_3_, but higher nitroalkanes perform poorly (Sartori et al., 1994). Mild reaction conditions allow for preparation of wide variety of oximes **7.7** and **7.8** in reasonable yields, but regioselectivities are often marginal (Scheme 7, Equation 2). *ortho*-Oximes **7.7** were obtained as pure *E*-forms, which was explained by coordination with metal cation, while *para*-isomers **7.8** were isolated as mixtures of *E*- and *Z*-isomers (Sartori et al., 1994). It should be pointed out, that less reactive arenes, such as mesitylene, toluene, benzene, and even chlorobenzene, could also be involved into this reaction, but in this case the products were obtained as mixtures of stereo- and regioisomers in poor yields (Kim et al., 1995).

Sometimes, oximes **7.10** generated *in situ* in the reaction of arenes with electrophilically activated nitroalkanes **7.9** can be involved into subsequent Beckmann rearrangement. The very first example of such reactivity was reported by Lambert, who demonstrated that nitromethane could react with benzene in the presence of AlCl_3_ to afford benzaldehyde oxime, which after acidic quench afforded aniline (**7.11**) (Scheme 7, Equation 3) (Lambert et al., 1949).

**Scheme 8:**
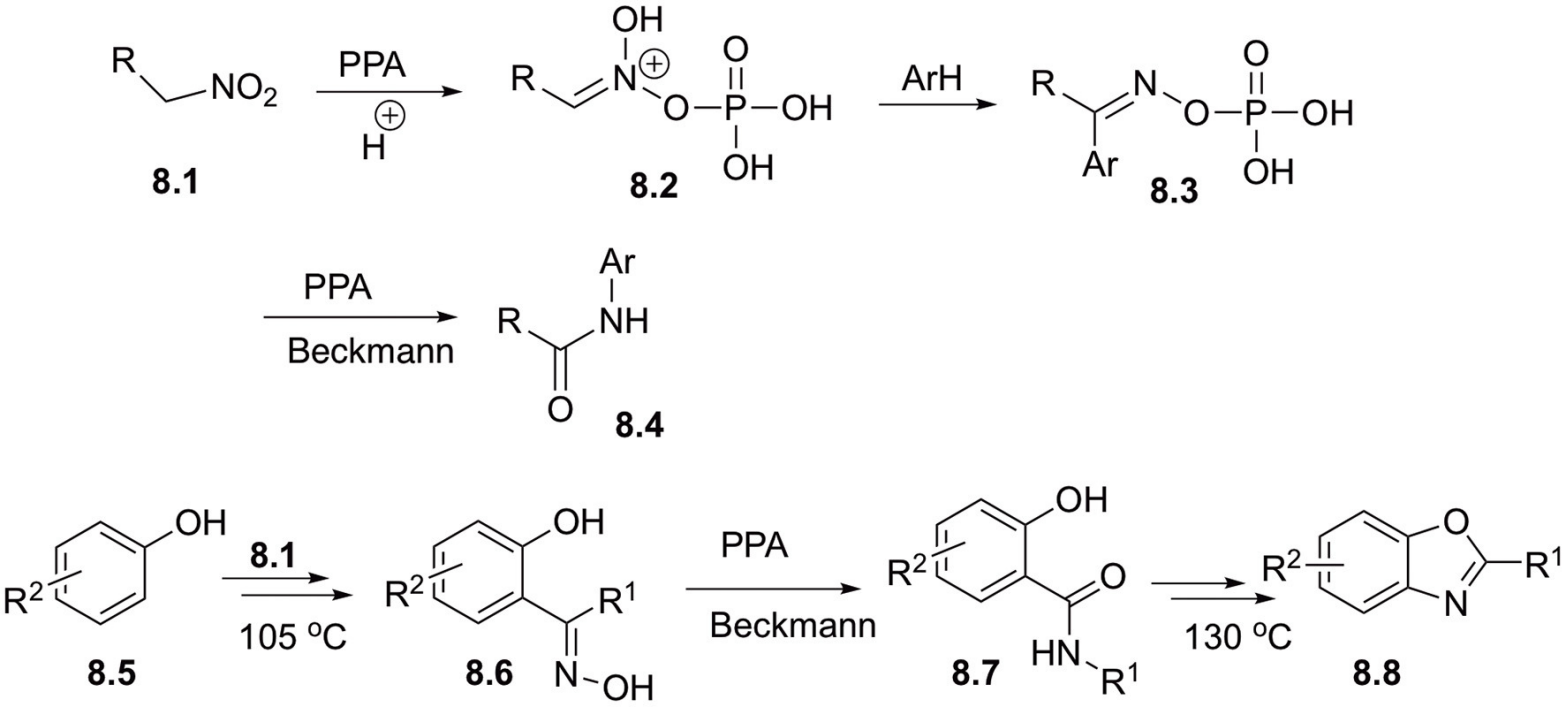
Reactions of primary nitroalkanes with arenes in polyphosphoric acid.

Electrophilic activation of nitroalkanes in combination with Beckmann rearrangement could be used for designing of very powerful synthetic approaches employing C-H functionalization strategies. Remarkably, it was shown that the outcome of this transformation strongly depends on the nature of nitroalkane employed. Aksenov demonstrated, that nitroethane and other higher primary nitroalkanes **8.1** in polyphosphoric acid (PPA) media exist in the form of electrophilic nitronates, stabilized in phosphorylated form **8.2**. These moieties could readily react with a variety of electron rich arenes and hetarenes to afford oxime derivatives **8.3**, which upon heating in PPA undergo further Beckmann rearrangement to yield carboxamides **8.4** (Scheme 8). Overall, this sequence of step works as highly efficient direct acetamidation of aromatic substrates (Aksenov et al., 2010). Interestingly, that the same reaction can be carried out in the presence of additional nucleophilic substituents to design a one-pot cascade process toward more complex heterocyclic scaffolds. For example, reaction with para-substituted phenols **8.5** provided oximes **8.6**, which after Beckmann rearrangement afforded carboxamide **8.7**. Subsequent condensation, involving ortho-hydroxyl substituent resulted in formation of benzoxazoles **8.8** in good yields (Scheme 8) (Aksenov N. A. et al., 2015). Related processes, featuring nucleophilic secondary nucleophilic attack with nitrogen atom to form pyrimidine ring was also reported (Aksenov et al., 2016).

**Scheme 9:**
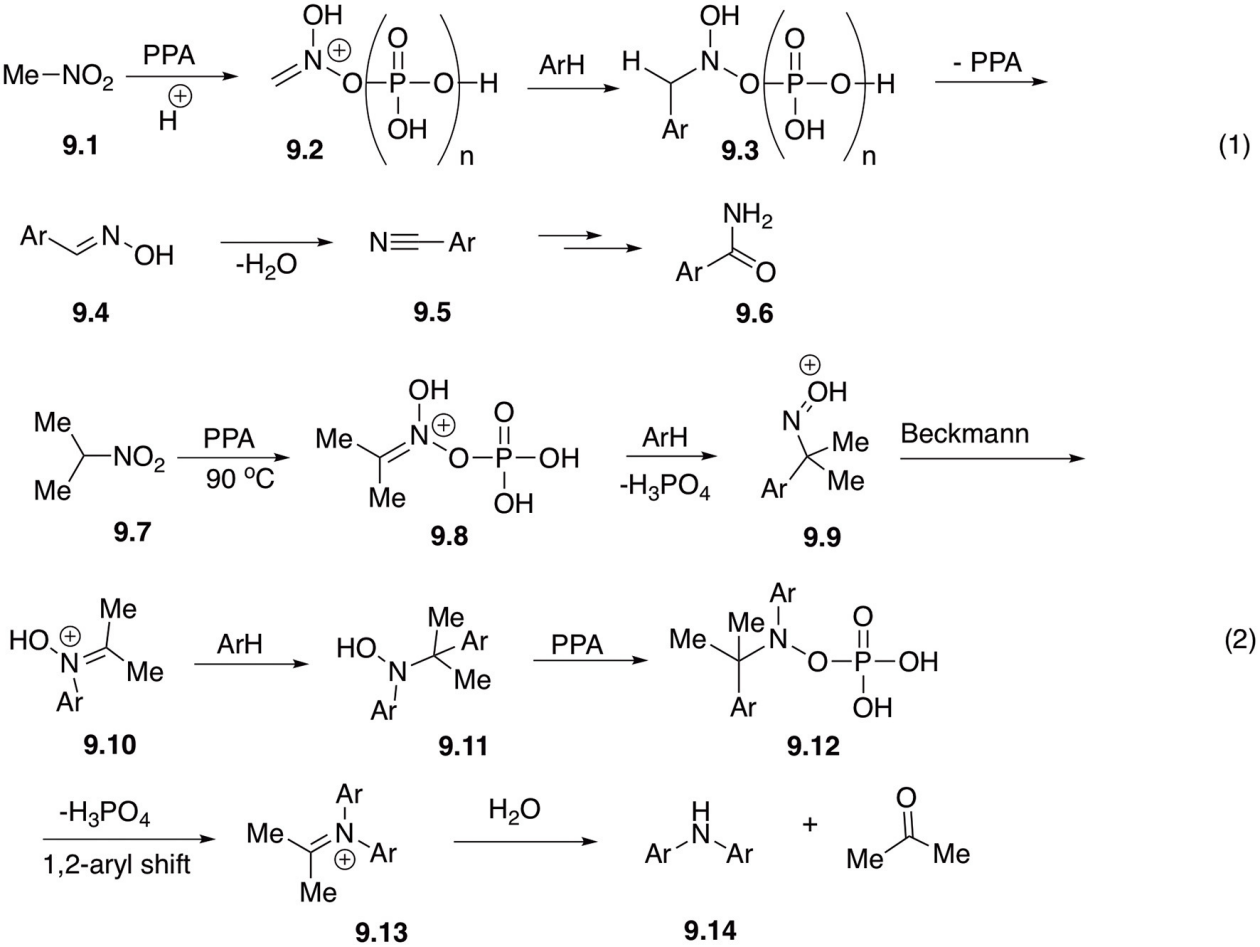
Reactions of nitromethane and secondary nitroalkanes with arenes in polyphosphoric acid.

Under the same reaction conditions process involving nitromethane **9.1** takes a different route (Scheme 1, Equation 1). Electrophilic nitronate **9.2** initially formed in PPA, first underwent similar reaction with electron-rich arenes to afford phosphorylated species **9.3**, and following elimination of phosphoric acid leading to formation of aldoxime **9.4**. The later species, however, does not undergo Beckmann rearrangement, losing a molecule of water instead and providing nitrile **9.5**. Finally, upon hydrolysis, primary amides **9.6** or the corresponding carboxylic acids were isolated (Scheme 9, Equation 1). Overall, instead of a new C-N bond formed in the reaction of higher primary nitroalkane homologs, here new C-C bond was introduced (Aksenov et al., 2012).

It was also shown that reactions involving secondary nitroalkanes, such as 2-nitropropane (**9.7**) proceed via a third alternative pathway (Scheme 9, Equation 2). Phosphorylated electrophilic nitronate species **9.8** first affords oxime **9.9**, which undergoes Beckmann rearrangement. Produced nitrone **9.10**, however, in this case lacks the α-CH bond and therefore cannot eliminate water to form a carboxamide. Instead, it experiences a second nucleophilic attack with arene to form hydroxylamine **9.11**. Phosphorylated form of this hydroxylamine (**9.12**) experiences a subsequent 1,2-aryl shift (mechanistically related to pinacole rearrangement), to afford iminium species **9.13**, which after hydrolytic quench produces diarylamine product **9.14** (Scheme 9, Equation 2) (Aksenov A. V. et al., 2015).

**Scheme 10:**
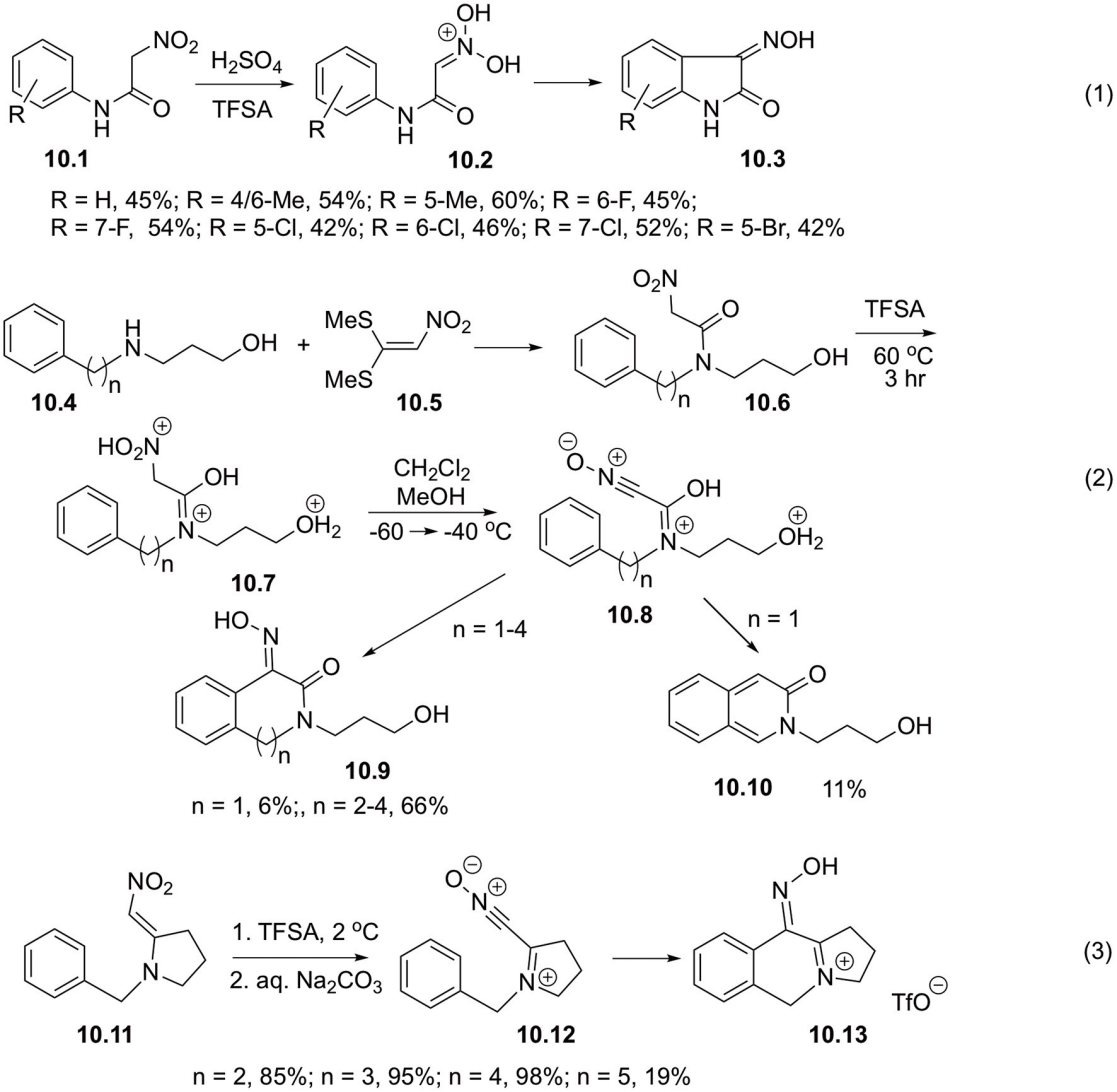
Cyclizations of nitroalkanes involving intramolecular reactions with electron-rich arenes.

It is worth mentioning, that related intramolecular reactions involving electrophilically activated could also be very efficient. Thus, it was shown that cyclization of 2-nitroacetanylides **10.1** obtained via base-mediated hydrolysis of 1-arylamino-1-methylthio-2-nitroethylenes readily proceeded at room temperature in the presence of sulfuric or triflic acid to afford oximes of indoline-2,3-diones **10.3**. It was stated, that protonated nitronates **10.2** serve as key intermediates in this transformation (Scheme 10, Equation 1) (Kearney et al., 1992a). It was also shown that intramolecular cyclizations of *N, N*-disubstituted nitroacetamides **10.6** bearing aryl substituent also occur in triflic acid. These starting materials can be generated in one-pot fashion in reaction between corresponding 3-(ω-arylalkylamino)propan-1-ols **10.4** and 1,1-bis(methylthio)-2-nitroethylene (**10.5**) (Scheme 10, Equation 2). To elucidate the mechanism of this transformation, the reaction was carried out at lowered temperature in sealed NMR tube. It was shown, that at 0°C nitroacetamide exists as triple-protonated species **10.7**, but elevation of the temperature leads to the formation of nitrile oxide **10.8** and subsequent cyclization into lactones **10.9** (Scheme 10, Equation 2). It should be pointed out, that formation of six-membered ring is normally accompanied by loss of oxime functionality and aromatization, leading to the formation of isoquinoline-3-ones **10.10** in poor yields (Scheme 10, Equation 2) (Fante et al., 2014a).

1-(ω-Phenylalkyl)-2-(nitromethylene)pyrrolidines **10.11** in triflic acid experience C,O-diprotonation with subsequent loss of water, leading to the formation of the corresponding nitrile oxides **10.12**. The latter species can be readily involved into intramolecular S_*E*_Ar reaction with phenyl substituent to afform tricyclic iminium species, isolated as triflic salts **10.13** in high yields (Scheme 10, Equation 3) (Fante et al., 2014b).

**Scheme 11:**
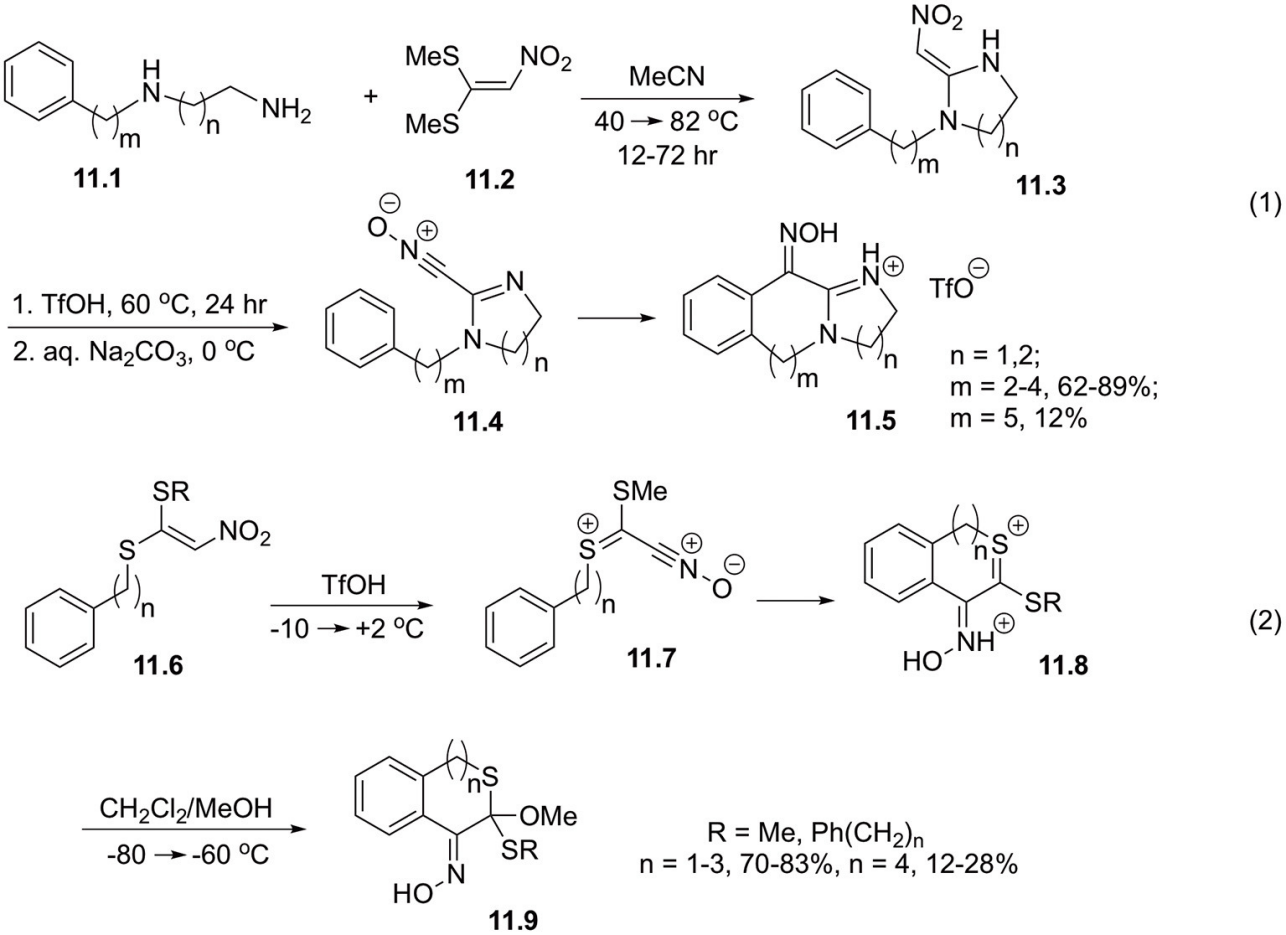
Cyclization involving 1,1-bis(methylthio)-2-nitroethylene.

Expectedly, 2-nitromethylene-1-(ω-phenylalkyl)imidazolidines and 2-nitromethylene-1-(ω-phenylalkyl)hexahydropyrimidines (**11.3**) showcase very similar reactivity. It should be pointed out, that the precursors for these cyclizations could be obtained via reaction of readily available 1,2- or 1,3-diamines **11.1** with 1,1-bis(methylthio)-2-nitroethylene **11.2** in boiling acetonitrlile. Cyclization of intermediate **11.4** takes place at 60°C in triflic acid, and provide oximes **11.5** in good yields for 7-9-membered rings. More challenging closure of 10-membered rings is much less efficient (Scheme 11, Equation 1) (Coustard et al., 2006).

It was also shown that derivatives of 1-(methylthio)-2-nitro-1-(phenylalkylthio)ethylene **11.6** in triflic acid provided nitrile oxide **11.7**, which underwent facile intramolecular cyclization affording intermediate dications **11.8**, which were detected by NMR spectroscopy. Low temperature quenching with methanol afforded cyclic oximes of isothiochromanones **11.9**, and their higher cyclic homologs. It was shown that ring sizes 6-8 could be assembled very efficiently, but larger rings were produced in marginal yields (Scheme 11, Equation 2) (Coustard, 2001).

**Scheme 12:**
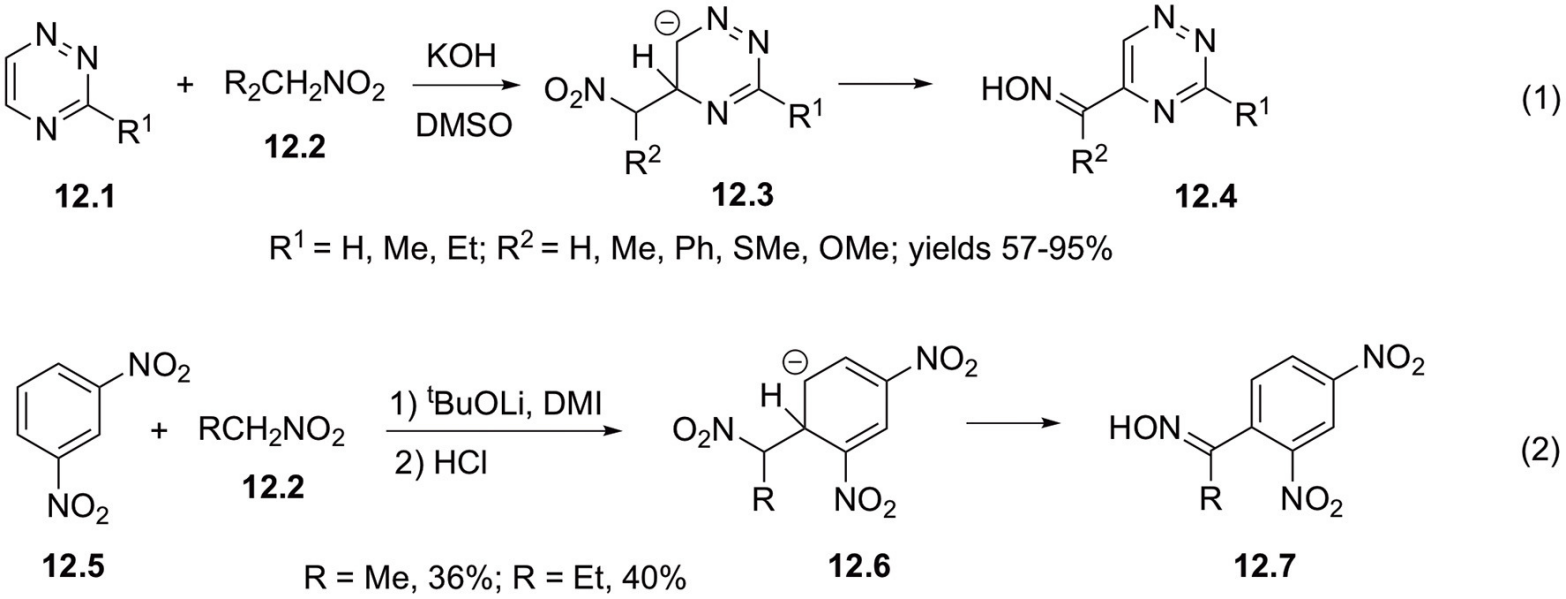
Vicarious substitution off involving electrophilic mitroalkanes.

Formally, vicarious substitution of hydrogen atom in hetarenes involving nitroalkanes in aci-form can also be regarded as related reaction. Thus, it was shown that anionic intermediate species **12.3** can be obtained in base-assisted interaction of nitroalkane **12.2** with triazines **12.1**, further converting into corresponding oximes **12.4** in good yields (Scheme 12, Equation 1) (Rykowski and Makosza, 1984). 1,3-Dinitrobenzene **12.5** also showed similar reactivity in the presence of strong bases affording anion 12.6, which subsequently transformed into oxime **12.7** (Scheme 12, Equation 2) (Kawakami and Suzuki, 1999).

**Scheme 13:**
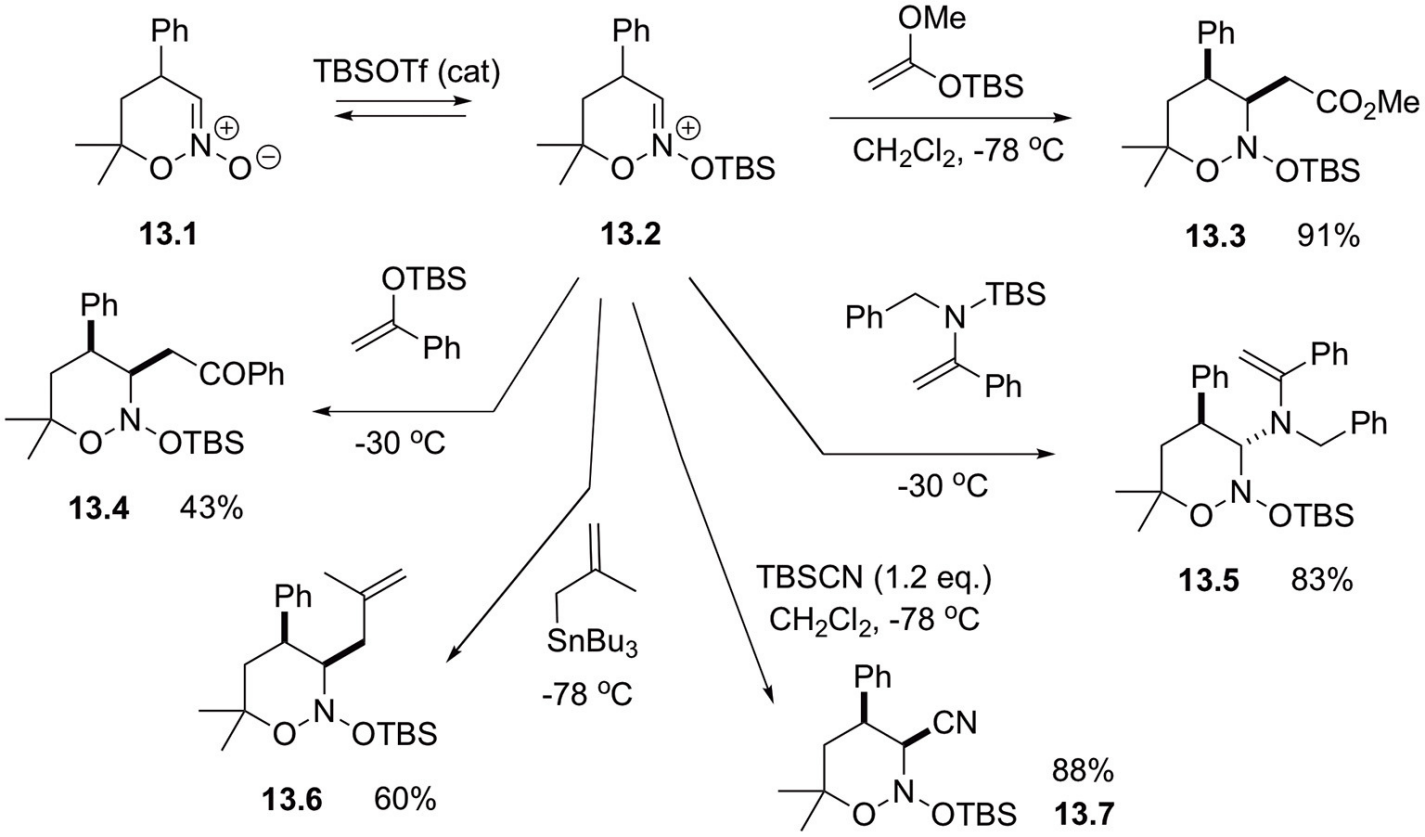
Alpha-alkylations of cyclic nitronates.

It should be pointed out that although cyclic nitronates are normally used as 1,3-dipoles, under certain conditions they could demonstrate electrophilic properties and can be readily engaged in the reactions with carbon-based nucleophiles. For example, cyclic species 13.1 was shown to react with TBSOTf to produce *in situ* highly reactive silated intermediate 13.2 susceptible for highly diastereoselective α-alkylation with ketene acetals, silylenol ethers, enamines, and allylstannanes (Scheme 13) (Smirnov et al., 2004). Reaction with silyl cyanides also was showcased to afford the corresponding α-cyanonitrozoacetal **13.7** in high yield (Scheme 13) (Mikhaylov et al., 2014).

**Scheme 14:**
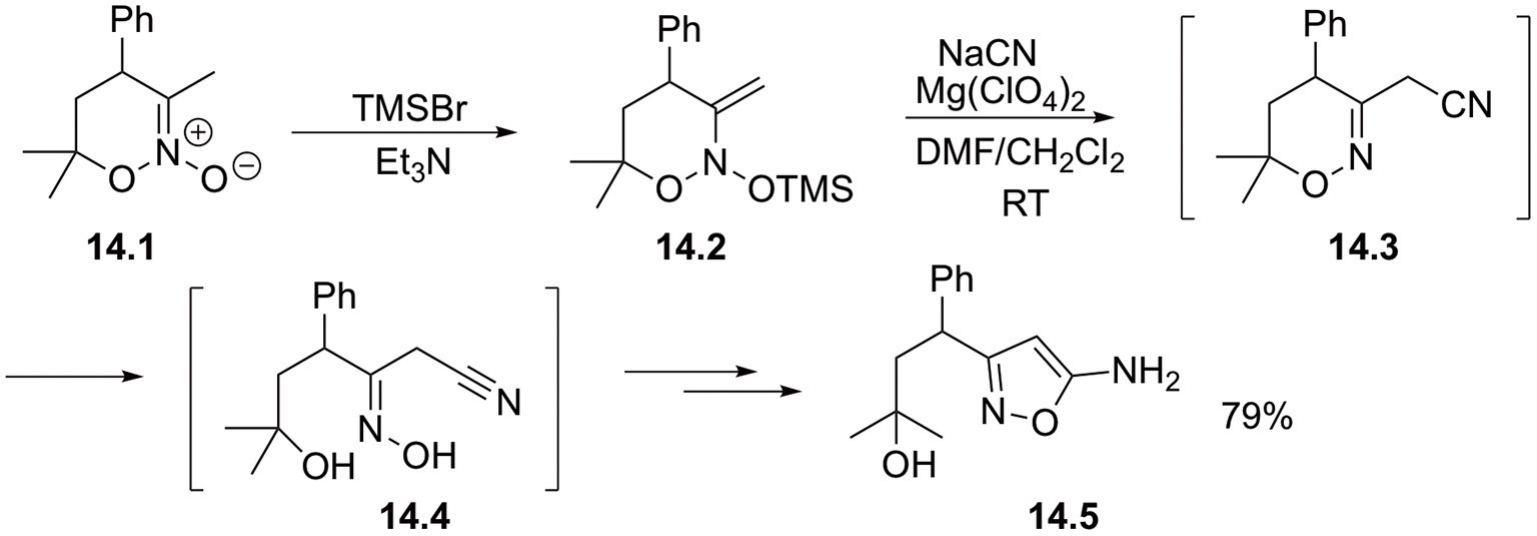
Beta-cyanation of cyclic nitronates.

It was also demonstrated that nitronates **14.1** bearing β-CH bonds afford *N*-silyloxyenamines **14.2** upon sylilation. Such species possess an electrophilic β-site and can react with alkali metal cyanide in the presence of Lewis acid. Desilation of the newly formed oxime function followed by nucleophilic cyclization to furnish isoxazole ring **14.5** in good yield (Scheme 14) (Ushakov et al., 2019).

In conclusion, electrophilic activation of nitroalkane species in the presence of acidic and basic reagents is presented. Although these compounds are typically employed after deprotonation at α-position as nucleophilic synthons, there is a possibility to exploit the umpolung method of activation. Nitronic acids and nitronates (aka aci-forms of nitroalkanes) demonstrate electrophilic behavior and enable reactions with a variety of carbon-based nucleophiles. These unusual reactions allow to design novel processes, involving C-C bond forming steps, C-H-functionalizations, ring closures, providing highly versatile tool for organic synthesis and medicinal chemistry applications.

## Author Contributions

All authors were equally responsible for preparation of this review, which included literature search and analysis, and the preparation of the manuscript.

### Conflict of Interest

The authors declare that the research was conducted in the absence of any commercial or financial relationships that could be construed as a potential conflict of interest.
